# Correction to “Spectral Map: Embedding Slow Kinetics in Collective Variables”

**DOI:** 10.1021/acs.jpclett.4c03457

**Published:** 2024-12-19

**Authors:** Jakub Rydzewski

In the original published article, the kernel density estimate below eq 3 was published as ϱ(**x**_*k*_) = ∑_*l*_κ(**z**_*k*_, **z**_*l*_). This equation should read instead ϱ(**z**_*k*_) = ∑_*l*_*g*(**z**_*k*_, **z**_*l*_). The typographical error does not change the results published in the article.

