# Supplementary material for: Spectral Map: Embedding Slow Kinetics in Collective Variables
Source: J Phys Chem Lett. 2023 Jun 1;14(22):5216–20. doi: 10.1021/acs.jpclett.3c01101 (PMC10258851; doi:10.1021/acs.jpclett.3c01101)
Supplement: Supplementary file 1 — jz3c01101_si_001.pdf [file jz3c01101_si_001.pdf]

Supporting Information: Spectral Map: Embedding Slow Kinetics in Collective Variables

Jakub Rydzewski*

Institute of Physics, Faculty of Physics, Astronomy and Informatics, Nicolaus Copernicus University, Grudziadzka 5, 87-100 Toruń, Poland

E-mail: jr@fizyka.umk.pl

Simulation Datasets

Molecular dynamics simulations are obtained from D.E. Shaw Research.[S1] For detailed simulation protocols, see Ref. S1 and its corresponding Supporting Online Material.

For chignolin, a ∼100-μs simulation in the NVT ensemble at a temperature of 340 K with conformations recorded every 200 ps is used. As a high-dimensional representation, pairwise Euclidean distances between the Cα atoms of CLN are employed, which amounts to $n = 45$ configuration variables. The training set consists of 5000 high-dimensional samples (extracted from the simulation every 2 ns). Spectral map is used to construct CVs for $k = 2$ metastable states.

For trp-cage, a ∼200-μs simulation in the NVT ensemble at a temperature of 290 K with conformations recorded every 200 ps is used. Pairwise Euclidean distances between the Cα atoms of trp-cage are employed; $n = 190$ configuration variables in total. The training set consists of 10000 samples (extracted from the simulation every 2 ns). Spectral map is used to construct CVs for $k = 2$ to 7 metastable states.

For BBA, a ∼200-μs simulation (the first trajectory from Ref. S1) in the NVT ensemble at a temperature of 325 K with conformations recorded every 200 ps is used. Pairwise Euclidean distances between the Cα atoms of BBA are employed; $n = 378$ configuration variables in total. The training set consists of 10000 samples (extracted from the simulation every 2 ns). Spectral map is used to construct CVs for $k = 3$ metastable states.

Spectral map is used to construct CVs for each system without any preprocessing of high-dimensional variables.

Target Mapping ξ_θ

A 5-layer neural network of size [n, 200, 100, 50, d] is used, where n and d are the number of variables in high- and low-dimensional representations, respectively. ReLU activation functions between each layer are employed. The Adam optimizer[S2] with default parameters and a learning rate of 0.0001 is used. The PyTorch library[S3] is used for the implementation.

During the training of the target mapping, each dataset is divided into data batches consisting of 100 samples. This data batch size used in spectral map for the construction of Markov transition matrices enables to perform many backpropagation steps for each epoch. This setup allows faster convergence to the maximal value of the spectral gap and reduces the computational time required for each eigendecomposition.

When creating anisotropic diffusion kernels to estimate Markov transition matrices, a scale constant ε of 1 is used for each batch. This constant only impacts the overall scale of CVs; thus, it can remain constant throughout the training process.

Fraction of Native Contacts

Following Ref. S4, to determine the fraction of native contacts, a list of pairs of Cα atoms (k, l) is used. If the distance between atoms k and l is less than 0.8 nm and the residues they belong to have a difference in sequence position greater than 3, they are considered in contact. The formula for calculating the fraction of native contacts is as follows:

$$q(\mathbf{x}) = \frac{1}{N} \sum_{kl} \frac{1}{1 + \exp[\alpha(r_{kl}(\mathbf{x}) - \gamma r^0_{kl})]}, \tag{1}$$

where N is the total number of native contacts, $r_{kl}(\mathbf{x})$ is the distance between Cα atoms k and l, r^0_{kl} is the distance between atoms k and l in the folded structure, $\alpha = 50$ nm^{-1}, and $\lambda = 1.5$.

Supporting Figures

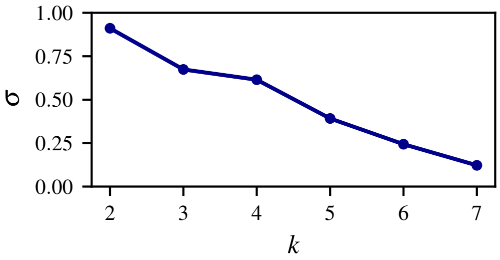

Figure S1: Spectral gaps ($\sigma = \lambda_{k-1} - \lambda_k$) obtained from training the target mapping for the trp-cage system calculated from $k = 2$ to 7 metastable states. Increasing the number of metastable states for this system results in a weaker timescale separation between slow and fast eigenvalues, negatively impacting the ability to capture slow CVs by spectral map. The maximal timescale separation is achieved for $k = 2$ metastable states, corresponding to the folded and unfolded ensembles of the trp-cage protein.

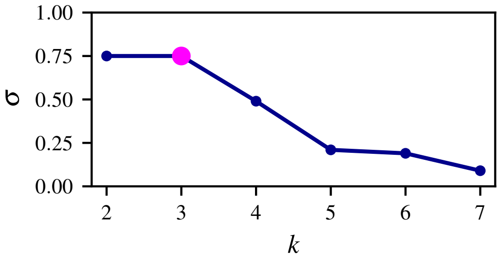

Figure S2: Spectral gaps ($\sigma = \lambda_{k-1} - \lambda_k$) obtained from the training of the target mapping for the BBA system calculated from $k = 2$ to 7 metastable states. In contrast to the trp-cage protein (Figure S1), a decline in the quality of timescale separation can be observed for the number of metastable states $k > 3$. As the spectral gap for $k = 3$ (magenta dot) is virtually equal to this of $k = 2$, the slow CVs of the BBA protein can be calculated for $k = 3$, providing more information about the slow dynamics of the system. For $k = 3$ metastable states, the system is represented by the folded, unfolded, and misfolded ensembles.

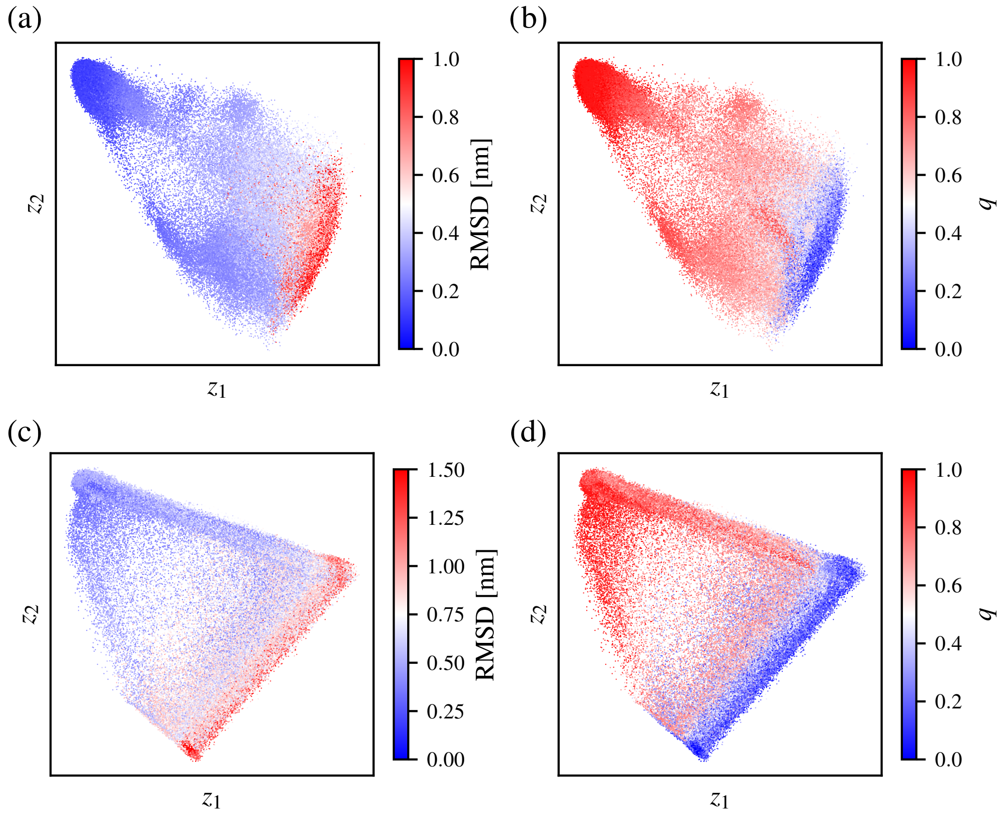

Figure S3: Comparison between CVs calculated using spectral map and standard variables used to characterize reversible folding processes for the (a-b) trp-cage and BBA (c-d) systems, considering Cα atoms. The RMSD values and the fraction of native contacts q (see eq 1) are computed in reference to the folded structures. For the trp-cage system, both standard variables can distinguish between the folded and unfolded states (a-b). In contrast, it is not possible for the BBA protein with three metastable states, i.e., while the folded state is easily classified, the unfolded and misfolded states are characterized by similar values (c-d).

References

(S1) Lindorff-Larsen, K.; Piana, S.; Dror, R. O.; Shaw, D. E. How Fast-Folding Proteins Fold. *Science* **2011**, *334*, 517–520.

(S2) Kingma, D. P.; Ba, J. Adam: A Method for Stochastic Optimization. *arXiv preprint arXiv:1412.6980* **2014**,

(S3) Paszke, A.; Gross, S.; Massa, F.; Lerer, A.; Bradbury, J.; Chanan, G.; Killeen, T.; Lin, Z.; Gimelshein, N.; Antiga, L.; others Pytorch: An Imperative Style, High-Performance Deep Learning Library. *Advances in Neural Information Processing Systems* **2019**, *32*.

(S4) Best, R. B.; Hummer, G.; Eaton, W. A. Native Contacts Determine Protein Folding Mechanisms in Atomistic Simulations. *Proc. Natl. Acad. Sci. U.S.A.* **2013**, *110*, 17874–17879.
